# Involvement of calpains in adult neurogenesis: implications for stroke

**DOI:** 10.3389/fncel.2015.00022

**Published:** 2015-02-04

**Authors:** Vanessa M. Machado, Maria I. Morte, Bruno P. Carreira, Maria M. Azevedo, Jiro Takano, Nobuhisa Iwata, Takaomi C. Saido, Hannelore Asmussen, Alan R. Horwitz, Caetana M. Carvalho, Inês M. Araújo

**Affiliations:** ^1^Regenerative Medicine Program, Department of Biomedical Sciences and Medicine, University of AlgarveFaro, Portugal; ^2^IBB-Institute for Biotechnology and Bioengineering, Center for Molecular and Structural Biomedicine, University of AlgarveFaro, Portugal; ^3^Center for Biomedical Research, CBMR, University of AlgarveFaro, Portugal; ^4^Center for Neuroscience and Cell Biology, University of CoimbraCoimbra, Portugal; ^5^Laboratory for Proteolytic Neuroscience, RIKEN Brain Science InstituteWako-shi, Saitama, Japan; ^6^Graduate School of Biomedical Sciences, Nagasaki UniversityNagasaki, Japan; ^7^Department of Cell Biology, University of Virginia School of MedicineCharlottesville, VA, USA

**Keywords:** calpains, calpastatin, hippocampus, migration, neurogenesis, proliferation, stroke, subventricular zone

## Abstract

Calpains are ubiquitous proteases involved in cell proliferation, adhesion and motility. In the brain, calpains have been associated with neuronal damage in both acute and neurodegenerative disorders, but their physiological function in the nervous system remains elusive. During brain ischemia, there is a large increase in the levels of intracellular calcium, leading to the activation of calpains. Inhibition of these proteases has been shown to reduce neuronal death in a variety of stroke models. On the other hand, after stroke, neural stem cells (NSC) increase their proliferation and newly formed neuroblasts migrate towards the site of injury. However, the process of forming new neurons after injury is not efficient and finding ways to improve it may help with recovery after lesion. Understanding the role of calpains in the process of neurogenesis may therefore open a new window for the treatment of stroke. We investigated the involvement of calpains in NSC proliferation and neuroblast migration in two highly neurogenic regions in the mouse brain, the dentate gyrus (DG) and the subventricular zone (SVZ). We used mice that lack calpastatin, the endogenous calpain inhibitor, and calpains were also modulated directly, using calpeptin, a pharmacological calpain inhibitor. Calpastatin deletion impaired both NSC proliferation and neuroblast migration. Calpain inhibition increased NSC proliferation, migration speed and migration distance in cells from the SVZ. Overall, our work suggests that calpains are important for neurogenesis and encourages further research on their neurogenic role. Prospective therapies targeting calpain activity may improve the formation of new neurons following stroke, in addition to affording neuroprotection.

## Introduction

Stroke is currently one of the main causes of brain damage and long-term disability. For this reason, therapeutic approaches aiming at repairing the lesion would highly beneficiate patients with this condition, in addition to providing neuroprotection against further damage. It is now well established that after the brain is formed new neurons are still produced throughout the adult mammalian life in discrete areas of the central nervous system, an event designated as neurogenesis (Gage, 2000). Neural stem cells (NSC), which can be found in the subventricular zone (SVZ) of the lateral ventricles and in the dentate gyrus (DG) of the hippocampus, constitute a pool of cells that exit these proliferative niches, migrate as neuroblasts towards regions where they are required and differentiate into mature neurons that integrate the neuronal circuitry (Aimone et al., 2014). Cells from the SVZ migrate through the rostral migratory stream (RMS) onto the olfactory bulbs, and are thought to be responsible for maintaining and reorganizing the interneuron system in the olfactory bulbs. In contrast, cells from the DG migrate shorter distances, from the subgranular zone (SGZ) into the granular zone (GZ) of the hippocampus, provide a substrate for additional brain plasticity and are crucial for spatial learning and memory (Imayoshi et al., 2008). Adult neurogenesis in rodents and other mammals is well established and has been widely characterized (for review, see Christian et al., 2014; Jessberger and Gage, 2014; Lim and Alvarez-Buylla, 2014). In humans, adult neurogenesis was characterized more recently, particularly in the hippocampus (Spalding et al., 2013) and in the striatum (Ernst et al., 2014).

Interestingly, stroke is followed by increased proliferation of NSC, and new neuroblasts migrate towards the site of injury (Arvidsson et al., 2002). In humans, there is also evidence of increased neurogenesis after stroke, even in older patients (Jin et al., 2006; Macas et al., 2006; Martí-Fàbregas et al., 2010; Popa-Wagner et al., 2014). Increased neurogenesis likely represents an endogenous attempt to regenerate cells lost in lesioned regions (Romanko et al., 2004). However, this process is limited and ineffective, since few neuroblasts reach the damaged region. In fact, the majority of the neuroblasts die before differentiating into functional mature neurons, thus failing the integration into the neuronal circuitry (Kaneko and Sawamoto, 2009; Ma et al., 2009). Only about 0.2% of dead neurons are replaced by newly generated neurons, which are not sufficient to compensate for neurological deficits (Arvidsson et al., 2002). The study of new strategies to promote post-injury repair may therefore focus in ways of enhancing neurogenesis in the adult brain.

After brain damage, the levels of intracellular calcium dramatically rise due to excitotoxicity, which leads to the activation of several proteases, including calpains (Neumar et al., 2001). Calpains are a family of calcium-dependent proteases that are involved in numerous processes, including cell adhesion and motility (cytoskeletal/membrane attachments), signal transduction pathways, cell cycle (proliferation), regulation of gene expression, apoptosis, and even long-term potentiation (for review, see Goll et al., 2003). The observed effects of calpain inhibition may be very different, depending on the cell type examined. In cell migration, for example, studies have shown that calpain inhibition impairs the migration ability of pancreatic beta-cells (Parnaud et al., 2005), vascular smooth muscle cells (Paulhe et al., 2001), T-cells (Rock et al., 2000), lung endothelial cells (Qiu et al., 2006), among others; calpain inhibition also increases the spreading ability of neutrophils (Lokuta et al., 2003). Moreover, there are also studies that show either a decrease (Croce et al., 1999) or an increase (Kuchay et al., 2012) in platelet spreading with the inhibition of calpains. Therefore, how calpains function in different cells and which are the signals that trigger their activity remain unclear.

In the brain, most of the studies regarding calpains focus on their involvement in neuronal damage. In animal models of brain ischemia, several calpain inhibitors have shown to be neuroprotective, being able to reduce neuronal damage caused by this pathology (Bartus et al., 1994; Hong et al., 1994; Li et al., 1998; Markgraf et al., 1998; Frederick et al., 2008; Koumura et al., 2008; Peng et al., 2011). However, there is little information on the physiological roles of calpains in cells from the central nervous system. Since calpains can influence cell proliferation and migration in other systems and NSC also share these functions, we investigated whether modulation of calpains can affect neurogenesis. We did this by analyzing the changes in NSC proliferation and neuroblast migration in the major neurogenic niches in the brain, the DG and the SVZ. Understanding the involvement of calpains in the modulation of NSC proliferation and neuroblast migration may help in the development of new strategies to improve post-injury brain repair.

## Results and discussion

**Figure 1:**
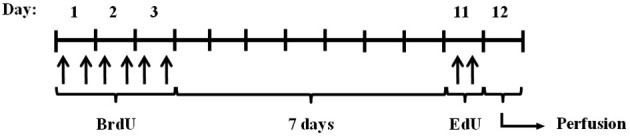
**Experimental procedure**. *Cast*^+/+^ and *Cast*^−/−^ mice were treated with BrdU for 3 days (every 12 h), and after 7 days with EdU (twice, 2 h apart). The animals were then sacrificed by transcardial perfusion, on day 12.

To study the effect of calpains on neurogenesis, we modulated calpain activity using inhibitors. One of the best approaches available to identify calpain functions *in vivo* is by altering the expression of calpastatin (Takano et al., 2005), the only known endogenous calpain inhibitor (Murachi, 1984). We did this by using mice lacking calpastatin (*Cast*^−/−^). We also used a pharmacological calpain inhibitor, calpeptin. Migration and proliferation of NSC in the hippocampus were traced with different thymidine analogs, as illustrated in Figure 1 and described in the methods section.

**Figure 2:**
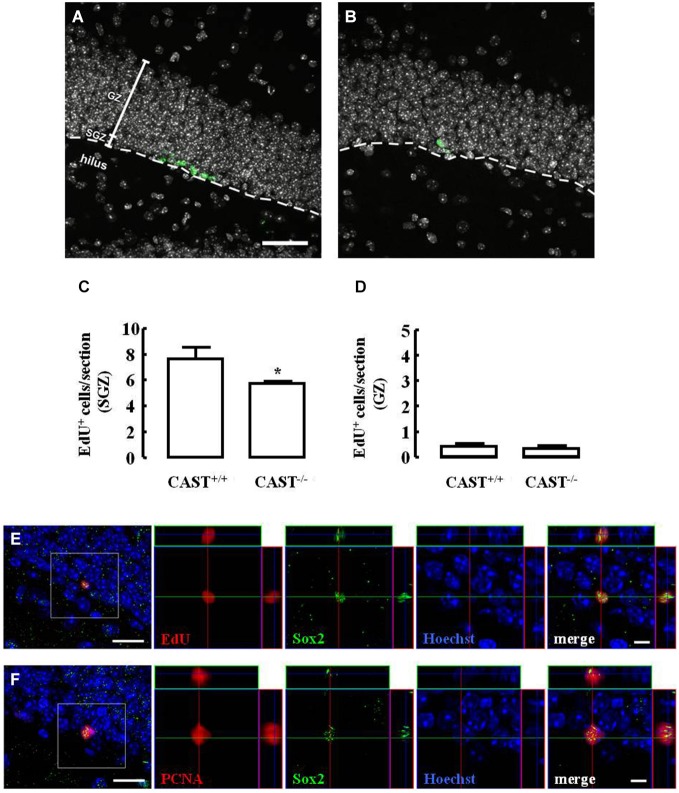
**Calpastatin deficiency impairs NSC proliferation in the SGZ**. Representative images from hippocampal brain sections of *Cast*^+/+^ **(A)** and *Cast*^−/−^ **(B)** mice, showing EdU-positive cells in green and nuclei, labeled with Hoechst 33342, in gray. Number of EdU-positive cells in the SGZ **(C)** and the GZ **(D)** of the hippocampus. Co-localization of EdU **(E)** or proliferating cell nuclear antigen (PCNA) **(F)**, in red, with the transcription factor Sox2, in green; nuclei are labeled with Hoechst 33342, in blue. Scale bars: 40 μm for **(A)** and **(B)**; 10 μm for the left panels of **(E)** and **(F)**; 5 μm for the right panels of **(E)** and **(F)**. Means ± SEM of at least 3 independent experiments. Two-tailed *t*-test, *p < 0.05 (significantly different from *Cast*^+/+^).

The effect of calpastatin deletion on the proliferation of NSC in the SGZ of the hippocampus was investigated by administering a thymidine analog, 5-ethynyl-2’-deoxyuridine (EdU), to both wild type (*Cast*^+/+^) and *Cast*^−/−^ mice on the day before their sacrifice. Incorporation of EdU in SGZ cells decreased in *Cast*^−/−^ mice (5.7 ± 0.2 cells/section, *p* = 0.0278) (Figures 2B,C), when compared to *Cast*^+/+^ mice (7.7 ± 0.9 cells/section) (Figures 2A,C). This shows that proliferation is affected when calpastatin is absent. In physiological conditions, NSC in the DG are formed in the SGZ, where they proliferate, and then migrate as neuroblasts into the GZ (Ming and Song, 2011). Since the number of EdU-positive cells in the GZ was not altered (0.4 ± 0.1 cells/section in *Cast*^+/+^ and 0.3 ± 0.1 cells/section in *Cast*^−/−^, *p* = 0.6738) (Figure 2D), the decreased number of EdU-positive cells observed in the SGZ was probably not due to enhanced migration into the GZ, supporting the idea of a decrease in the proliferation caused by loss of calpastatin. Since Sox2 controls NSC maintenance in the hippocampus (Favaro et al., 2009; Ehm et al., 2010), co-localization of this transcription factor with either EdU (Figure 2E) or proliferating cell nuclear antigen (PCNA; Figure 2F) was performed to show that dividing cells are NSC.

As previously reported, *Cast*^−/−^ mice do not present increased intrinsic calpain activity (Takano et al., 2005), but the absence of calpastatin is expected to deregulate calpain activity, by loss of inhibition, when calpain activation is needed during cell proliferation. Suppression of calpain activity has been reported to reduce the proliferation of different types of cells, such as vascular smooth muscle cells (Ariyoshi et al., 1998), Chinese hamster ovary cell colonies (Xu and Mellgren, 2002), osteoblasts (Shimada et al., 2008), chondrocytes (Kashiwagi et al., 2010) and lung endothelial cells (Qiu et al., 2006). More recently, the inhibition of calpains was also shown to decrease the proliferation of mouse NSC lines (Santos et al., 2012). Different cells may act differently under different stimuli, so the exact mechanisms through which calpains affect cell proliferation must be studied in further detail, to clarify these differences.

**Figure 3:**
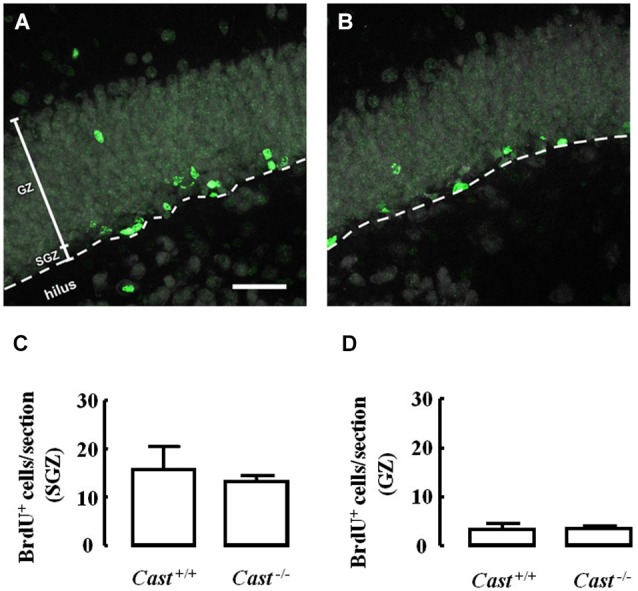
**Calpastatin deficiency does not alter the distribution of BrdU-positive cells in the DG**. Representative images from hippocampal brain sections of *Cast*^+/+^ **(A)** and *Cast*^−/−^ **(B)** mice, showing BrdU-positive cells in green and nuclei, labeled with NeuN, in gray. Number of BrdU-positive cells in the SGZ **(C)** and the GZ **(D)** of the hippocampus. Scale bar: 40 μm. Means ± SEM of 5 independent experiments. Two-tailed *t*-test, *p* > 0.05.

**Figure 4:**
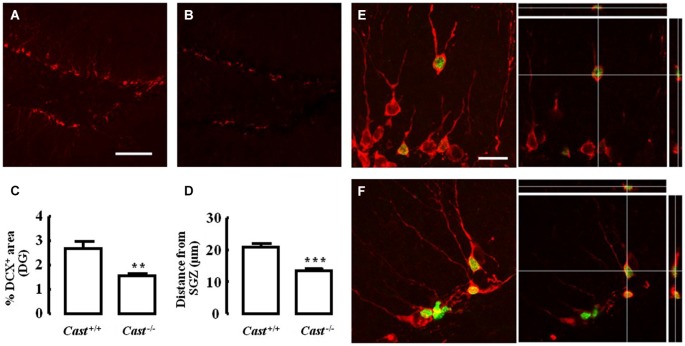
**Calpastatin deficiency decreases DCX immunoreactivity and migration distance of newborn cells in the DG**. Representative images from hippocampal brain sections of *Cast*^+/+^ **(A)** and **(E)** and *Cast*^−/−^ **(B)** and **(F)** mice, showing migrating neuroblasts in red, labeled for DCX, and BrdU-positive cells in green. Percentage of DCX immunoreactivity in the DG **(C)**. Distance migrated from the SGZ by the BrdU-positive/DCX-positive cells present in the GZ **(D)**. Co-localization of BrdU and DCX is shown in the right panels of **(E)** and **(F)**. Scale bars: 100 μm for **(A)** and **(B)**; 20 μm for **(E)** and **(F)**. Means ± SEM of at least 3 independent experiments. Two-tailed *t*-test, **p < 0.01 and ***p < 0.001 (significantly different from *Cast*^+/+^).

**Figure 5:**
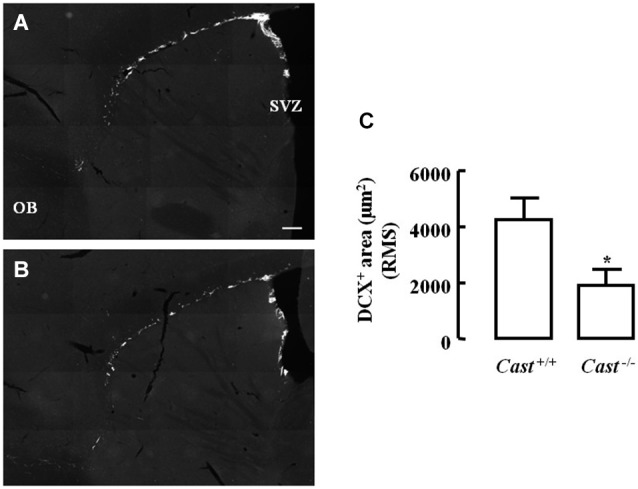
**Calpastatin deficiency decreases DCX immunoreactivity in the RMS**. Representative images from sagittal striatal brain sections of *Cast*^+/+^ **(A)** and *Cast*^−/−^ **(B)**, showing migrating neuroblasts in gray, labeled for DCX. OB, olfactory bulb. DCX-positive area in the RMS **(C)**. Scale bar: 200 μm. Means ± SEM of 5 independent experiments. Two-tailed *t*-test, *p < 0.05 (significantly different from *Cast*^+/+^).

To analyze whether calpastatin deletion affected the migration of newly formed NSC in the DG, another thymidine analog, 5-bromo-2’-deoxyuridine (BrdU), was administered to the mice on days 9–11 prior to their sacrifice. The analysis of migration in the DG was performed by three different methods: distribution of BrdU-positive cells in the SGZ and the GZ, doublecortin (DCX) immunoreactivity, and distance of BrdU-positive/DCX-positive cells from the SGZ into the GZ. The number of BrdU-positive cells in *Cast*^+/+^ mice (Figure 3A) was 15.7 ± 4.8 cells/section in the SGZ (Figure 3C) and 3.3 ± 1.3 cells/section in the GZ (Figure 3D). In *Cast*^−/−^ mice (Figure 3B), we observed a similar distribution of BrdU-positive cells in the DG, with 13.1 ± 1.3 cells/section in the SGZ (*p* = 0.6184, Figure 3C) and 3.4 ± 0.5 cells/section in the GZ (*p* = 0.9329, Figure 3D). While the analysis of BrdU distribution did not suggest a decreased number of newborn cells migrating into the GZ of *Cast*^−/−^ mice, the overall neuroblast migration in the DG was reduced, as determined by measuring the percentage of DCX-positive area (1.5 ± 0.1% in *Cast*^−/−^ and 2.7 ± 0.3% in *Cast*^+/+^, *p* = 0.0049) (Figures 4A,B,C). Moreover, BrdU-positive/DCX-positive cells, which are indicative of migratory newborn cells, presented shorter migration distances in *Cast*^−/−^ mice (13.5 ± 0.6 μm, *p* = 0.0003) (Figures 4D,F), comparing to *Cast*^+/+^ mice (20.8 ± 1.1 μm) (Figures 4D,E). These results suggest an impairment of neuroblast migration in the DG of calpastatin-deficient mice, in addition to decreased NSC proliferation. Moreover, this effect on neuroblast migration was not limited to the DG, we also observed an impairment of cell migration in the RMS of *Cast*^−/−^ mice (1899.9 ± 575.3 μm^2^, *p* = 0.0039) (Figures 5B,C), when compared to *Cast*^+/+^ mice (4259.2 ± 764.8 μm^2^) (Figures 5A,C).

Calpains have been shown to be involved in the migration of a variety of cell types, particularly by regulating cell spreading, speed and adherence, focal contact formation, filopodial and lamellipodial protusion formation and chemokinesis, with the involvement of signaling proteins such as Rac1, RhoA, Cdc42 and phospholipase C (Potter et al., 1998; Croce et al., 1999; Paulhe et al., 2001; Lokuta et al., 2003; Parnaud et al., 2005; Kuchay et al., 2012). Even though calpain inhibition results in decreased migration for most cell types already studied, there are reports showing that calpain inhibition increases the spreading ability of neutrophils (Lokuta et al., 2003) and, more recently, platelets (Kuchay et al., 2012). Although further research is needed to better understand the physiological roles of calpains, the results here presented suggest that promoting calpain activation may impair the formation of newborn neuronal cells, by interfering with the first stages of neurogenesis.

**Figure 6:**
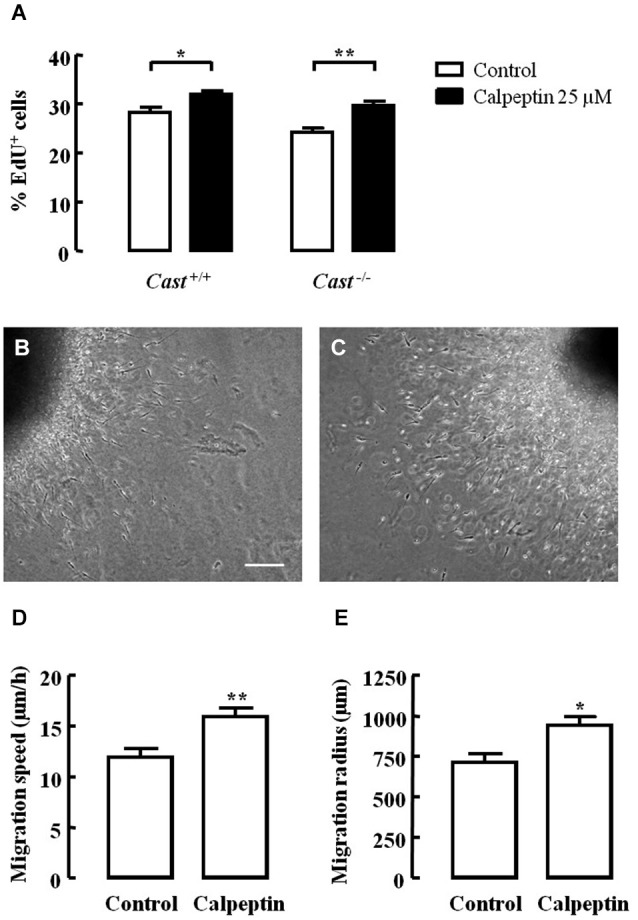
**Calpain inhibition increases NSC proliferation and cell migration in *Cast*^+/+^ cells and rescues cell proliferation in *Cast*^−/−^ cells**. Percentage of living cells positive for EdU **(A)**. Representative images of SVZ explants untreated **(B)** or with calpeptin 25 μM **(C)**. Migration speed **(D)** and radius **(E)** of cells migrating out of the SVZ explants. Means ± SEM of at least 3 independent experiments. Two-way ANOVA (Bonferroni’s post-test)** (A)** or two-tailed *t*-test **(D)** and **(E)**, *p < 0.05 and **p < 0.01 (significantly different from control). Scale bar: 100 μm.

Excitotoxicity after stroke activates calpains, and several studies have already demonstrated that calpain inhibition is effective in reducing brain damage in animal models of this pathology. If calpain inhibition could enhance post-injury neurogenesis in addition to providing neuroprotection, it could be a potential therapeutic option to reduce brain damage after stroke. In this context, we investigated the effect of calpain inhibition on NSC proliferation and neuroblast migration in culture, using the calpain inhibitor calpeptin. Cell proliferation, as analyzed by EdU incorporation (Figure 6A), was lower in *Cast*^−/−^ cells (24.2 ± 0.9%) than in *Cast*^+/+^ cells (28.4 ± 1.0%), which is consistent with our *in vivo* observations. Calpeptin reversed this effect (29.7 ± 0.8%, *p* < 0.01), indicating that calpains may in fact be mediating cell proliferation. Moreover, calpeptin also increased cell proliferation in *Cast*^+/+^ cells (32.0 ± 0.7%, *p* < 0.05). With the goal of enhancing post-injury neurogenesis, this slight rather than high increase in cell proliferation is actually preferable, since it lowers the risk of undifferentiated mass growth, while still somewhat increasing the number of newborn cells that may later on replace lost neurons. Nevertheless, increasing neuroblast migration and consequent neuronal differentiation, integration and survival is important for successfully improving brain repair after stroke.

Finally, we also assessed cell migration in SVZ explants isolated from wild type mice. Calpeptin (Figure 6C) increased both the migration speed (16.0 ± 0.8 μm/h, *p* = 0.0063) (Figure 6D) and the migration distance (943.0 ± 51.9 μm, *p* = 0.0120) (Figure 6E) of cells leaving the explants, when compared to untreated cells (migration speed 12.0 ± 0.8 μm/h; migration distance 715.0 ± 50.2 μm) (Figures 6B,D,E). This translates into another advantage for the potential use of calpain inhibition to enhance post-injury neurogenesis, i.e., improving migration. However, studies on how calpain inhibition may affect neuronal differentiation, integration and survival are still needed in order to corroborate the idea of using calpain inhibitors to treat brain damage after stroke.

Calpains are key players in the neuronal damage that occurs after stroke (Bano and Nicotera, 2007; Bevers and Neumar, 2008). For this reason, some of the strategies that have been developed in order to limit neuronal death after ischemic lesion have focused on interfering with calpain function. Several calpain inhibitors were already shown to be neuroprotective in animal models of brain ischemia (Bartus et al., 1994; Hong et al., 1994; Li et al., 1998; Markgraf et al., 1998; Frederick et al., 2008; Koumura et al., 2008; Peng et al., 2011). Furthermore, overexpression of calpastatin (Cao et al., 2007) and calpain silencing (Bevers et al., 2010) have also been shown to be effective in reducing neuronal death caused by stroke. The results presented here show that the lack of calpastatin hinders proliferation of NSC in the SGZ of the hippocampus, as well as migration of newborn cells into the GZ and in the RMS of adult mice. Moreover, we show that calpain inhibition increases SVZ-derived NSC proliferation and cell migration in wild type cells. We propose that, in addition to their neuroprotective effect, the use of calpain inhibitors results in an enhancement of post-injury brain repair. This constitutes an added benefit for potential clinical application for the treatment of stroke. Neuroprotection afforded by inhibition of calpains was also observed in other pathologies in which calpains contribute to the neuronal damage. These include traumatic brain injury (Saatman et al., 1996; Schoch et al., 2012), Alzheimer’s disease (Rao et al., 2008), Parkinson’s disease (Crocker et al., 2003), spinal cord injury (Ray et al., 2000), diabetic retinopathy (Shanab et al., 2012), acute optic neuritis (Das et al., 2013) and optic nerve crush (Araujo Couto et al., 2004).

Stem cells offer a promising approach for brain repair after stroke. In addition to the enhancement of endogenous neurogenesis, other approaches recently reported include transplantation after stroke of NSC obtained from human fetal brain (Mine et al., 2013), induced pluripotent stem cells (Tornero et al., 2013) or stem cells from other origins (for review, see Lindvall and Kokaia, 2011; Hermann et al., 2014; Ikegame et al., 2014). Directly inducing the conversion of astrocytes into neurogenic cells is also being regarded as a possibility for post-injury brain repair (Niu et al., 2013; Magnusson et al., 2014). However, these approaches are currently hampered by limited cell survival due to the unfavorable conditions present in the lesioned areas. Such unfavorable conditions seem to be particularly worsened with age, conditioning the outcome of brain repair, partly by reducing the natural increase of endogenous neurogenesis (Popa-Wagner et al., 2014). Nonetheless, stem cell therapies have also shown to at least partially improve recovery after stroke in aged rodents (Jin et al., 2010; Zhang et al., 2013; Balseanu et al., 2014; Tatarishvili et al., 2014). Thus, combined therapies pose a promising approach to enhance post-injury brain repair, both in young and aged brains.

Overall, inhibiting calpains may improve the outcome of brain repair strategies based on cell therapy by both limiting neuronal damage and enhancing neurogenesis.

## Material and methods

### Animals

Twenty-week old calpastatin knock-out mice (*Cast*^−/−^, *n* = 5) (Takano et al., 2005), in a C57Bl6 background, and their wild type littermates (*Cast*^+/+^, *n* = 5), were used in this study. The mice were kept in our animal facilities, in a room with controlled temperature (21 ± 1°C) and humidity (55%), with food and water *ad libitum* in a 12 h dark:light cycle. All experiments were performed in accordance with institutional and European guidelines (2010/63/EU) for the care and use of laboratory animals.

### Labeling of dividing cells with thymidine analogs

The experimental procedure was conducted as illustrated in Figure 1. Briefly, the animals (5 *Cast*^−/−^ and 5 *Cast*^+/+^) were treated for 3 days with BrdU (Sigma Aldrich, St Louis, MO, USA), through i.p. injections of 50 mg/kg every 12 h. After 7 days, EdU was administrated (Invitrogen, Paisley, UK), through i.p. injections of 50 mg/kg, twice, 2 h apart. This was performed in order to assess cell migration and cell proliferation, respectively. On day 12, the mice were perfused transcardially with 0.9 % NaCl followed by 4 % paraformaldehyde. The brains were removed and coronal hippocampal sections and sagittal striatal sections were obtained by cryosectioning (30 μm thick, in 6-series). The sections were stored in an antifreeze solution, at 4°C.

### Immunohistochemistry and detection of thymidine analogs

Free-floating coronal hippocampal sections were processed for immunohistochemistry against BrdU, DCX and neuronal nuclei (NeuN), or double-labeled against PCNA and Sox2, or EdU and Sox2. DNA denaturation for BrdU staining was performed by treating the sections with 1 M HCl for 20 min at 65°C. Antigen retrieval for Sox2 or PCNA stainings was performed by treating the sections with 10 mM citric acid, pH 6.0, for 20 min at 95°C. The sections were then blocked for 1 h with 5 % normal horse or goat serum (Vector Laboratories Inc., Burlingame, CA, USA), respectively, in 0.25 % Triton X-100 in 0.01 M PBS. Slices were incubated with the primary antibodies, mouse anti-BrdU (1:80; DAKO, Glostrup, Denmark) or rat anti-BrdU (1:50, AbD Serotec, Oxford, UK), goat anti-DCX (1:400; Santa Cruz Biotechnology, Santa Cruz, CA, USA), mouse anti-NeuN (1:200; Millipore Corporation, Billerica, MA, USA), mouse anti-PCNA (1:50; Santa Cruz Biotechnology, Santa Cruz, CA, USA) or rabbit anti-Sox2 (1:250; Millipore Corporation, Billerica, MA, USA) for 48 h at 4°C. The sections were then incubated with the correspondent secondary antibody (1:200), in 2 % block (normal horse or goat serum, accordingly), conjugated with Alexa Fluor 488, Alexa Fluor 594 or Alexa Fluor 633 (Invitrogen, Paisley, UK), for 2 h in the dark, at room temperature. Labeling for EdU was performed by click-chemistry with Alexa Fluor 488 or 594 azide, according to the manufacturer instructions (Invitrogen, Paisley, UK) and, with the exception of the BrdU staining, nuclei were stained with Hoechst 33342 (2 μg/ml; Invitrogen, Paisley, UK) for 10 min. Free-floating sagittal striatal sections were stained against DCX. The sections were mounted on gelatin-coated slides with DAKO fluorescence mounting medium (DAKO, Glostrup, Denmark).

### Analysis of incorporation of BrdU and EdU

BrdU-positive and EdU-positive cells in the SGZ, the first layer of cells adjacent to the hilus, and in the GZ of 5 mid sections of the hippocampus (spanning 180 μm of the dorsal hippocampus) were counted for each animal (Liu et al., 1998; Brunson et al., 2001; Salazar-Colocho et al., 2008; Kim et al., 2009; Carreira et al., 2010), directly under an epifluorescence microscope (Axioskop 2 Plus, Zeiss, Jena, Germany), by one blinded observer. Cell counting was carried out in both upper and lower blades of the DG.

### DCX immunoreactivity and migration measurements in the DG

DCX immunoreactivity in the DG and migration distances of BrdU-positive/DCX-positive cells in the GZ were determined in images acquired in a laser scanning microscope (LSM 510 Meta, Zeiss, Jena, Germany), using ImageJ (version 1.43u, National Institutes of Health, Bethesda, MD, USA) with the LSM Toolbox Plugin. The quantification of the DCX-positive area was performed using a threshold analysis in 5 mid sections of the hippocampus of each animal. This consisted in defining the optimal threshold for stained *vs*. non stained cells and calculating the area stained with DCX (Komitova et al., 2005; Carreira et al., 2010). The migration measurements, in turn, were performed in a total of approximately 30 cells from 5 images acquired for each *Cast*^+/+^ or *Cast*^−/−^ mouse, by determining the distance between the nucleus of the cell and the boundary between the SGZ and the hilus, perpendicularly to that delimitation.

### DCX immunoreactivity in the RMS

DCX immunoreactivity in the RMS was determined using ImageJ (version 1.43u, National Institutes of Health, Bethesda, MD, USA), in images acquired in an inverted microscope (Axio Observer Z1, Zeiss, Jena, Germany), using the Mosaix module from the AxioVision software Rel. 4.8.2 (Zeiss, Jena, Germany), in order to capture the entire RMS on each section. DCX-positive area was measured for each image in 3 boxes of 250 μm × 250 μm, randomly placed along the length of the RMS, a method similar to what was previously described (Kuhn et al., 1997).

### NSC proliferation in SVZ cultures

NSC were isolated from the SVZ of P0-3 *Cast*^+/+^ and *Cast*^−/−^ mice and maintained in culture, as previously described (Morte et al., 2013). Dissociated NSC (*n* = 3) were plated on coverslips coated with 0.1 mg/ml poly-L-lysine (Sigma Aldrich, St Louis, MO, USA) until 60–70% confluency was reached, and then treated with calpeptin 25 μM (Tocris Bioscience, Bristol, UK) for 6 h (untreated cells were used as controls). The cells were kept with EdU 10 μM for the last 4 h before fixation with 4% paraformaldehyde/4% sucrose. EdU incorporation was processed using a commercially available kit (Click-iT®EdU Alexa Fluor®488 HCS Assay, Invitrogen, Paisley, UK) and nuclei were stained with 1 μg/ml Hoechst 33342, for 5 min. EdU-positive cells were counted in images acquired in an Axio Imager Z2 microscope (Zeiss, Jena, Germany).

### NSC migration SVZ explants

SVZ explants were prepared from wild type C57Bl6 mice (P5–7) and cultured for 72 h in 70% Matrigel (BD Biosciences, San Jose, CA, USA) in CCM1 medium (Hyclone, Logan, UT, USA) supplemented with 1% Pen/Strep and 1% B27 (Invitrogen, Paisley, UK) (Wichterle et al., 1997; de Chevigny et al., 2006), in the presence (*n* = 5) or absence (*n* = 8) of calpeptin 25 μM. Migration distances were measured in 5 explants per culture and migrating cells were imaged for 3 h, at 3 min intervals, under an Eclipse TE200 inverted microscope (Nikon Corporation, Tokyo, Japan), using the Metamorph 6.3v1 software (Molecular Devices, Sunnyvale, CA, USA), in order to calculate the migration speed of cells leaving the explants.

### Statistical analysis

The data are presented as means ± SEM. Statistical significance was determined using a two-tailed *t*-test or a two-way ANOVA, as indicated in the figure legends, using GraphPad Prism 5 software. Differences were considered significant when *p* < 0.05.

## Author contributions

Vanessa M. Machado, Inês M. Araújo: Conception and design of the work, acquisition, analysis, interpretation of data, drafting of manuscript;

Maria I. Morte, Bruno P. Carreira, Maria M. Azevedo: Acquisition, analysis, interpretation of data;

Jiro Takano, Nobuhisa Iwata, Takaomi C. Saido: Conception and design of the work, analysis, interpretation of data;

Hannelore Asmussen, Alan R. Horwitz: Conception and design of the work, acquisition, analysis, interpretation of data;

Caetana M. Carvalho: Conception and design of the work, interpretation of data.

## Conflict of interest statement

The authors declare that the research was conducted in the absence of any commercial or financial relationships that could be construed as a potential conflict of interest.

## Abbreviations

BrdU: 5-bromo-2’-deoxyuridine
*Cast*^+/+^: wild type
*Cast*^−/−^: calpastatin knock-out
DCX: doublecortin
DG: dentate gyrus
EdU: 5-ethynyl-2’-deoxyuridine
GZ: granular zone
NeuN: neuronal nuclei
NSC: neural stem cells
PCNA: proliferating cell nuclear antigen
RMS: rostral migratory stream
SGZ: subgranular zone
SVZ: subventricular zone.
